# Droplet evaporation residue indicating SARS-COV-2 survivability on surfaces

**DOI:** 10.1063/5.0038562

**Published:** 2021-01-15

**Authors:** Zilong He, Siyao Shao, Jiaqi Li, S. Santosh Kumar, J. B. Sokoloff, Jiarong Hong

**Affiliations:** 1 Department of Mechanical Engineering, University of Minnesota, Minneapolis, Minnesota 55455, USA; 2 Saint Anthony Falls Laboratory, University of Minnesota, Minneapolis, Minnesota 55414, USA; 3 Department of Physics, Northeastern University, Boston, Massachusetts 02115, USA; 4 Department of Physics, Florida Atlantic University, Boca Raton, Florida 33431, USA

## Abstract

We conducted a systematic investigation of droplet evaporation on different surfaces. We found that droplets formed even with distilled water do not disappear with evaporation but instead shrink to a residue of a few micrometers lasting over 24 h. The residue formation process differs across surfaces and humidity levels. Specifically, under 40% relative humidity, 80% of droplets form residues on plastic and uncoated and coated glass, while less than 20% form on stainless steel and none on copper. The formation of residues and their variability are explained by modeling the evaporation process considering the presence of nonvolatile solutes on substrates and substrate thermal conductivity. Such variability is consistent with the survivability of SARS-CoV-2 measured on these surfaces. We hypothesize that these long-lasting microscale residues can potentially insulate the virus against environmental changes, allowing them to survive and remain infectious for extended durations.

## I. INTRODUCTION

The ongoing COVID-19 pandemic has infected more than 80 million people as of now, causing major disruption to the global economy and social order. It has been well accepted that the severe acute respiratory syndrome coronavirus-2 (SARS-CoV-2) is causing the disease and can be transmitted through the contact of virus-laden respiratory droplets on surfaces. Particularly, studies have found much higher concentration of SARS-CoV-2 RNA deposited as droplets on surfaces in hospitals rather than as aerosols,^1,2^ pointing to the importance of investigating the virus survivability on surfaces. As reported by two recent experiments,^3,4^ SARS-CoV-2 has a long survival time on different surfaces and can remain viable under different temperature and humidity levels. Specifically, Chin *et al.*^3^ investigated the stability of SARS-CoV-2 deposited as droplets on ten surfaces at 60% relative humidity (RH) with variation in temperatures and found the virus to be more stable on smooth surfaces (e.g., glass and plastic), remaining viable for up to two to four days, respectively, with survival time decreasing at higher temperatures. Similarly, van Doremalen *et al.*^4^ found virus survival time on four surfaces, at 40% RH, to vary from ∼7 h on copper to more than three days on plastic (polypropylene). However, so far only Bhardwaj and Agrawal^5,6^ have provided some physical mechanisms to explain the long survival times, the large variation between the different surface materials tested, as well as the impact of environmental changes on surface transmission. In particular, they attributed the long survival time of the viruses to the shielding of a thin film (400 nm–600 nm height with the wetted radius of 1 mm∼4 mm) surrounding the viruses. Such mechanisms, related to the droplet evaporation process, can be critical for understanding the carriage and transmission of SARS-CoV-2 as summarized in a recent review paper.^7^ Here, we hypothesize that the evaporation characteristics of respiratory droplets may indicate SARS-CoV-2 survivability on different surfaces and under different humidity and temperature conditions. In the literature, studies of droplet evaporation on surfaces typically involve seeded particles and focus on particle pattern formation for various applications such as inkjet/3D printing and manufacturing self-assembled structures.^8^

Only one study investigated the evaporation of ultrapure water droplets on hydrophobic substrates that generates submicron residues.^9^ There is no systematic experimental study of such water droplet evaporation on different surfaces of interest and making the connection between virus transmission and droplet evaporation. Therefore, we report a systematic experiment to assess the evaporation process of distilled water droplets on surfaces. This paper is structured as follows: Sec. II describes the experimental setup, equipment, and measurement methods. In Sec. III, we first present our experimental observation of the evaporation of distilled water on different surfaces, which reveals the formation of residues and its variation on different surfaces. Subsequently, we develop a physical evaporation model to explain the phenomenon of residue formation. We further investigate the stability and durability of residues and the humidity effects on the residue fraction and final residue size. Finally, the conclusion and further discussion are provided in Sec. IV.

## II. METHODOLOGY

Our evaporation experiments are conducted using distilled water droplets with the deposited droplet size ranging from 5 *μ*m to 100 *µ*m, within the range of respiratory droplets generated by human breathing and speaking.^10^ Distilled water is selected instead of respiratory droplets to minimize the variability of droplet chemical content on our test results. Additionally, test surfaces are chosen to match those used in the work of Chin *et al.*^3^ and van Doremalen *et al.*^4^ The water droplets are generated using distilled water with a TSI 9302 nebulizer operated at an input pressure of 138 kPa, which produces a 5.7 l/min output rate of droplets (mean diameter ∼6.4 *µ*m) that coagulate on the surface to produce a wide range of droplet sizes. Five different surface samples, including a Fisher Scientific microscope glass slide, a glass slide coated with RainX hydrophobic coating, plastic (3M polypropylene tape), copper (Hillman copper sheet), and 304 stainless steel samples, are selected for testing under an ambient temperature of 22 °C and humidity varying between 25% and 60% RH. The samples are placed with the test side facing up on an inverted microscope connected with a Flare CMOS camera (2048 pixel × 1024 pixel sensor size) sampling at 30 fps. We use the nebulizer to generate droplets on the substrate and imaged them simultaneously under 10× magnification (1.21 × 0.64 mm^2^ field of view at 0.59 *μ*m/pixel resolution) to capture the evaporation of liquid droplets and the formation of the residues. The size of evaporating droplets at each time step and the corresponding residues are extracted from the 10× microscopic images manually using ImageJ, where the size is defined as the area-equivalent diameter. We conduct residue removability tests for each substrate through heating and wiping. For the former, we treat each surface with a heat gun (temperature of 60 °C at the surface) for 60 s and observe, both qualitatively and quantitatively, the change in the residue concentration. For the latter, we wipe the surfaces with a Kimtech wipe for ∼10 s with minimal pressure. Finally, we test the long-term stability and durability of the residues on all surfaces (except copper) by capturing images at 10× magnification for 24 h, at 1 h increments, in an environment with a relatively stable temperature (22 °C) and humidity (40% RH).

## III. EXPRIMENTAL RESULTS

### A. Residues form on surfaces from droplet evaporation

**FIG. 1.**
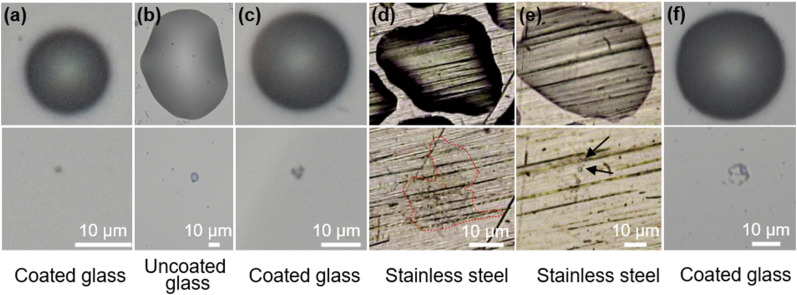
A gallery of original droplets (upper) and their corresponding residues (lower) indicating the various morphologies of residues formed. Single residues form by (a) non-pinning droplets evaporating on a coated glass surface, (b) pinned droplets evaporating on an uncoated glass surface, (c) film recoil of pinned droplets, and (d) contact pinned evaporation on stainless steel forming a large area of residue (marked by outline). Multiple residues form due to (e) roughness induced film breakup on a stainless steel surface (with arrows marking the individual residues) or (f) surface tension induced film breakup on a coated glass substrate.

We found that during evaporation, droplets on the tested surfaces first shrink in height (constant contact radius mode) and then in diameter (constant contact angle mode) to form a thin liquid film, leaving behind residues of different types on the order of micrometers, as illustrated in Fig. 1. We either obtain a single residue, most likely a thin film or droplet, or multiple residues formed by breakup of a thin film. Single residues form through evaporation on a glass surface, both in the absence of surface adhesion for a hydrophobic surface [Fig. 1(a) and Video S1] and on a hydrophilic surface with strong adhesion [Fig. 1(b) and Video S2]. Near the end of evaporation on a coated glass substrate, sometimes the thin liquid film recoils due to the effect of surface tension, leaving behind a larger concentrated residue in the middle [Fig. 1(c) and Video S3]. Alternatively, on a stainless steel surface, a strong hydrophilic behavior of the evaporating droplet results in a large area of thin film residue [Fig. 1(d) and Video S4]. We do observe similar thin films on copper substrates but with a thickness much smaller than for stainless steel. Our approach is thus unable to fully quantify the residue size on copper surfaces due to the weaker signal inherent to such thin films at this humidity level. Finally, the formation of multiple residues is often through breakup of a pinned film due to surface roughness, e.g., on stainless steel [Fig. 1(e) and Video S5], or surface tension instabilities, e.g., on coated glass [Fig. 1(f) and Video S6].

**FIG. 2.**
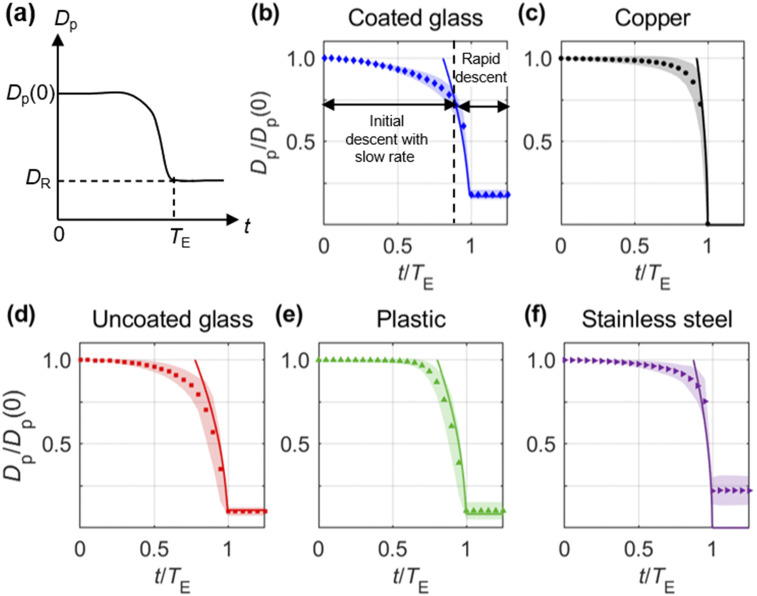
(a) Schematic of the evaporation curve illustrating the variation of droplet size with time. *D*_p_(0) is the initial droplet diameter, and *T*_E_ is the time at which the droplet forms the residue of size *D*_R_. Normalized evaporation curves on (b) coated glass, (c) copper, (d) uncoated glass, (e) plastic, and (f) stainless steel surfaces at a temperature of 22 °C and humidity of 40% RH. The normalized evaporation curves are calculated by averaging 100 individual droplets evaporating on each surface. The measured time varying sizes from the images are used as sample points to generate a continuous evaporation curve at discrete time steps through piecewise Hermite polynomial interpolation. The standard deviation indicating the differences between the sampled droplets is presented as the shading around each data point and the evaporation model by the solid line.

To quantify the droplet evaporation process, we measure the wetted diameter (*D*_p_) as a function of time (*t*) for the different surfaces [Figs. 2(b)–2(f)]. We define *D*_p_ as the area-equivalent diameter of the droplet to enable comparisons between non-spherical and spherical shapes observed. The initial droplet size *D*_p_(0) is measured at the start of evaporation when the droplet begins to change in size or height. The evaporation time *T*_E_ is defined as the time at which the droplet shrinks to the residue size *D*_R_, i.e., *D*_p_(*T*_E_) = *D*_R_. In cases where the droplet disappears completely, we set *D*_p_(*T*_E_) = 0, while for cases with multiple residues, we measure *D*_R_ that is defined as the root mean square of the individual residue sizes. To characterize the general evaporation trend of droplets of different sizes, the evaporation curves are normalized using *D*_p_(0) and *T*_E_ corresponding to each droplet.

For the coated glass surface [Fig. 2(b)], the evaporation curve exhibits an initial slow rate of change in size over a duration of ∼0.8*T*_E_, followed by a rapid descent to form the final residue of about 18% of *D*_p_(0). Compared with the coated glass surface, the evaporation curves for the other surfaces show a similar trend in general [Figs. 2(c)–2(f)]. However, the evaporation rate and residue size vary among different surfaces, depending on the surface properties including wettability, roughness, and thermal conductivity. Specifically, coated glass that has strong hydrophobicity and smoothness presents the highest initial evaporation rate. The metal surfaces (i.e., copper and stainless steel) with higher thermal conductivity exhibit a steeper change in size near the end of evaporation, compared to plastic and both glass surfaces with low thermal conductivity. The copper substrate does not yield any resolvable residue at 40% RH, while the residues for the other surfaces fall within the range of 9%–22% of *D*_p_(0). The rougher surfaces such as plastic and stainless steel show larger variation in residue size compared to the smoother glass surfaces.

**FIG. 3.**
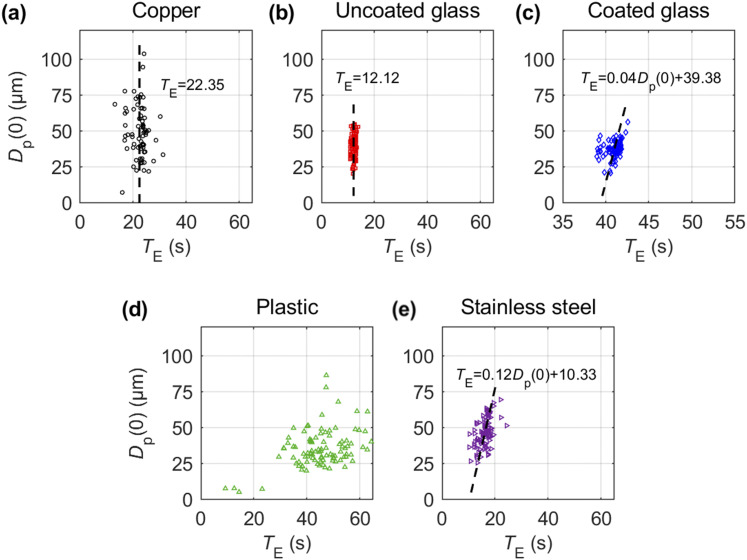
Variation in droplet evaporation time (*T*_E_) as a function of initial droplet size *D*_p_(0) for (a) copper, (b) uncoated glass, (c) coated glass, (d) plastic, and (e) stainless steel surfaces. Dashed line indicates the linear least-squares fit between *D*_p_(0) and *T*_E_ for coated glass and stainless steel and mean *T*_E_ for copper and coated glass. Data are not fitted for plastic since the data appear to be highly scattered with no clear trend observed.

The initial droplet diameter *D*_p_(0) and evaporation time *T*_E_ yield approximately a linear relationship under our experimental conditions for all surfaces except copper and uncoated glass for which *T*_E_ shows little dependence on *D*_p_(0) (Fig. 3). The slope varies strongly across the different surfaces, from ∼0.12 for stainless steel to ∼0.04 for the coated glass surface. Interestingly, our measurements on the copper surface show no clear dependence between the droplet size and evaporation time, possibly due to the high thermal conductivity influencing the evaporation process. The plastic surface, on the other hand, does not show a clear trend in the measurements and also takes the longest time for evaporation, on average, followed by the coated glass. Such trends compare favorably to lower evaporation rates expected on hydrophobic surfaces due to the smaller surface area exhibited by the droplet. In contrast, all hydrophilic surfaces measure evaporation times that are approximately half of the hydrophobic glass, with the uncoated glass showing even faster evaporation. The large scatter in the data for copper, stainless steel, and plastic cases can be attributed to the variation in droplet shapes and sizes as well as the variety of residue types formed on those surfaces, which points to the presence of multiple evaporation mechanisms. Surfaces with minimal variation in droplet residue type, i.e., both glass surfaces, show the least amount scatter from the linear trend.

### B. Physical mechanism of residue formation

Hu and Larson proposed an approximate equation for evaluating the rate of evaporation of water.^11^ However, this model implicitly assumes that the temperature at the liquid–gas interface is the same as the ambient temperature. This assumption fails when the substrates have low thermal conductivity and thin thickness (below ∼150 μm) as shown by the experiments of David *et al.*^12^ and Diddens *et al.*^13^ and theoretical analyses by Sefiane and Bennacer^14^ and Schofield *et al.*^15^ Specifically, Schofield *et al.* pointed out that the lifetime of droplets is significantly extended on substrates with low thermal conductivity.^15^

There are many research studies reporting the residues formed by phase segregation^16^ and crystallization^17^ from multicomponent sessile droplet evaporation, and Diddens^18^ used a finite element method to model this process. There is only one paper discussing the formation of microscale residues from pure water evaporation by He and Darhuber,^9^ which suggests that this phenomenon is a result of deliquescence by ionic compounds in the photoresist substrate. However, such a mechanism cannot explain the observations from the current experiment using substrates without similar ionic compounds. Bhardwaj and Agrawal^6^ developed a model using disjoining and Laplace pressures to explain the long survival time with the assumption that the height of the drop is 400 nm–600 nm and the wetted radius is 1 mm∼4 mm. However, we found that residue droplets can yield wetted radius orders of magnitude smaller than their assumption. We attribute the formation of residues to the presence of nonvolatile solutes on substrates that gradually dissolve into the droplet near the contact line during the evaporation. The dissolution of such nonvolatile content slows down and eventually ceases evaporation, leaving residues on substrates. A physical model of this evaporation process [Eq. (1)] is proposed by including the effects of both non-volatile solute^19^ and substrate conductivity^14^ on the quasi steady evaporation rate equation proposed by Hu and Larson,^11^

$$\begin{array}{ll} \dot{m} & =\frac{\rho dV}{dt}=-\frac{\pi D_{p}D}{2}\left(1-\frac{\phi \left(t\right)D_{0}^{3}}{D_{p}{\left(t\right)}^{3}}-RH\right)C_{s}\left(0.27\theta^{2}+1.3\right)\times M, \end{array}$$ (1)

where *ρ* is the density, *D*_p_ is the wetted diameter of the droplet, *V* is the volume, *D* is the diffusion coefficient of water vapor, *ϕ* is the volume fraction of the solute (evaluated by the Nernst and Brunner equation), *D*_0_ is the initial droplet wetted diameter, RH is the relative humidity (higher humidity corresponds to higher RH, which slows down the evaporation process), *C*_*s*_ is the saturation vapor concentration at the liquid–gas interface, *θ* is the contact angle (note that *θ* is larger for superhydrophobic substrates; the effect of wettability is incorporated in the current model from the contact angle), and *M* is the relative evaporating ratio, which is defined as the ratio of *C*_*s*_ to saturated vapor concentration in ambient air. It is between 0.267 and 1 at 22 °C, coupling substrate conductivity and evaporative cooling of the droplet.^14,19^ For substrates with high thermal conductivity such as copper, *M* equals to 1 as the temperature at the liquid–gas interface is the same as the ambient temperature, and it decreases with the substrate conductivity as the temperature at the liquid–gas interface is lower than the ambient temperature due to the evaporative cooling effect and low substrate conductivity. The lowest possible *M* is calculated by dividing the saturated vapor concentration at 0 °C to *C*_*s*_ since we do not observe the freezing effect on experiments. The dissolution of the nonvolatile solute is described by the Nernst and Brunner equation^20^

$$\frac{dC\left(t\right)}{dt}=\frac{D_{s}A\left(t\right)}{V\left(t\right)h_{d}}\left(C_{sn}-C\left(t\right)\right),$$ (2)

where *C* is the concentration of solute inside the droplet, *D*_*s*_ is the diffusion coefficient of the solute in the solvent, *h*_*d*_ is the thickness of the diffusion layer, *A* is the area near contact line, *V* is the volume of the droplet, and *C*_*sn*_ is the solubility of the solute.

Note that the droplet shape is controlled by the Bond number *Bo* = *ρgD*_p_*h*_0_/2*σ* (ratio of gravitational force to surface tension) and the capillary number $Ca=\mu \bar{u_{r}}/\sigma$ (ratio of viscous to capillary forces), where *ρ* is the fluid density, g is the gravitational constant, *h*_0_ is the initial height of the droplet, *σ* is the air–water surface tension, *μ* is the liquid viscosity, and $\bar{u_{r}}$ is the average radial velocity induced by evaporation. In our experiments, wetted diameter *D*_p_ ∼ 0.1 mm and *h*_0_ ∼ 0.01 mm, and $\bar{u_{r}}∼1\;\mu m/s$.^11^ Based on this information, we estimate that *Bo* ∼ 10^−4^ and *Ca* ∼ 10^−8^. Therefore, we can treat the droplet shape as a spherical cap with the volume given by $V=\pi h\left(3{D_{p}}^{2}+D_{p}\;\tan{\left(\theta /2\right)}^{2}\right)/24$. The values for *D* and *C*_*s*_ are evaluated by equations from the work of Kumar and Bhardwaj,^21^ while the contact angle *θ* is taken from prior studies with similar experimental conditions.^22–24^ Sharma *et al.* experimentally showed that when droplets impact on the substrates with particles, the particles migrate naturally to the contact line, supporting our hypothesis that the dissolution happens near the contact line.^25^ The concentration of the solute calculated is converted into the volume fraction *ϕ* using *ϕ* = *C*/*ρ*_*solute*_/(*C*/*ρ*_*solute*_ + *V*). The properties of the solute (*C*_*sn*_, *ρ*_*solute*_, *D*_*s*_, and *h*_*d*_) are estimated based on the values of sodium chloride in water. These values are validated by setting *M* = 1 for copper and comparing the model results with the experiments, which yields a 11% maximum relative error. *M* is estimated by our numerical model for other substrates in the range of 0.267–1. Netz derived an analytic expression for the residue size as a function of the solute volume fraction and the relative humidity $D_{R}=D_{p}\left(0\right){\left(\phi_{0}/\left(1-RH\right)\right)}^{1/3}$, where *ϕ*_0_ is the initial solute concentration.^19^

We compare the time scale of dissolution (*τ*_*dis*_ ∼ *dD*_p_/*D*_s_) and evaporation time found from Refs. 14 and 19 Using the above assumptions and substitutions, Eq. (1) can be written as

$$\begin{array}{ll} \frac{dD_{p}}{dt} & =M\times \frac{24D}{\rho \;\tan\frac{\theta }{2}\left(3+tan^{2}\frac{\theta }{2}\right)D_{p}}\left(1-\frac{\phi \left(t\right)D_{0}^{3}}{D_{p}{\left(t\right)}^{3}}-RH\right)\\ & \;\times \;C_{s}\left(0.27\theta^{2}+1.3\right). \end{array}$$ (3)

The volume fraction of the solute is allowed to increase only in the limit where dissolution is much faster than evaporation (*dt* ≫ *τ*_*dis*_). The coupled equations [Eqs. (2) and (3)] are solved numerically in MATLAB with a constant contact angle assumption except for coated glass, which we assume with a linear decreasing contact angle starting from ∼0.8*T*_F_,^26^ the results for which are shown in Figs. 2(b)–2(f). The time of the constant contact radius mode is obtained from our experiments. The slow evaporation process on low conductivity substrates and high RH environment allows more solute to dissolve, leading to larger residues.

The maximum relative error is 15% for coated glass, 15% for uncoated glass, 13% for plastic, and 11% for stainless steel. Our model has three limitations. It fails to predict the fraction and the eventual decay of residues on different substrates. In addition, the contact angle starts to slowly decrease as the droplet shrinks to a residue, making our model deviate from experiments. Finally, our model predicts that there are no residues on stainless steel, contradicting to our experiments. This can be explained by the fact that due to the porosity of the substrate, the evaporation near *T*_E_ on stainless steel does not follow our assumptions that the droplet shape is a spherical cap (Videos S4 and S5).

### C. Residues show long-term stability and durability

**FIG. 4.**
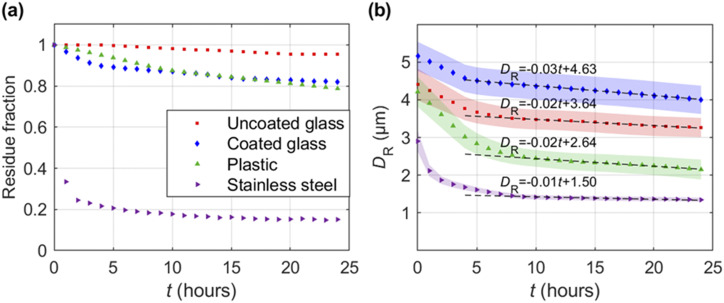
Long term stability of residues on various test surfaces measured at a temperature of 22 °C and humidity of 40% RH. (a) Residue fraction as a function of time on each substrate. (b) Average area-equivalent diameter *D*_R_ of residues sampled over the same duration with the shaded region representing the standard error, and the dashed lines indicate the linear least-squares fit conducted over a range of *t* near the end of each dataset where a linear trend can be clearly observed, from above ∼5 h for coated glass to data above ∼8 h for the remaining.

The resolvable residues exhibit a stability in number and size for a period of 24 h, as shown in Fig. 4. Specifically, the percentage of residues that remain, referred to as the residue fraction, decays gradually with time for all surfaces except for stainless steel, which displays a sharp decline at the beginning, reaching a plateau at ∼15% potentially due to the relatively higher thermal conductivity and a larger contact area associated with surface roughness. The uncoated glass retains the highest residue fraction (∼95%), while the coated glass and plastic both yield a lower fraction of ∼80% after 24 h. The drop in residue fraction can be attributed to the evaporation of smaller residues present on these surfaces, as indicated by the larger variability in residue size seen in Fig. 4. The average residue size [Fig. 4(b)] for all the surfaces show a relatively larger decrease within the first few hours, followed by an almost linear decay with a very shallow slope (−0.01 *µ*m/h to −0.03 *µ*m/h) at longer durations, indicating that their survival time could extend well beyond 24 h. The mechanism of the long survival time of residues was discussed by Bhardwaj and Agrawal.^6^ They showed that when the height of the droplets approaches the submicrometer scale, the evaporating rate is governed by disjoining and Laplace pressures inside the film, leading to slow evaporation.

Once formed, these residues show strong durability even under fluctuations of ambient temperature and humidity. They can stay on plastic and glass surfaces even after the surfaces are treated with a heat gun for 60 s at a temperature of ∼60 °C (measured at the surface), while the same treatment removes more than ∼90% of residues on stainless steel, possibly due to its higher thermal conductivity. In comparison, we found that wiping is more effective for residue removal across all surfaces (applying Kimtech wipes for 10 s can remove >95% of the residues).

### D. Humidity influences formation of residues

We found that the residue formation process is strongly influenced by the ambient humidity. The increase in humidity from 25% RH to 60% RH leads to an increase in the fraction of residue forming droplets, with coated glass increasing from 55% to 90%, plastic from 5% to 30%, and copper from 0% to 15% (i.e., no residues to residues at higher humidity). On the other hand, the stainless steel and uncoated glass surfaces show no significant change in the fraction of residues with the increase in humidity (remaining at ∼55% for stainless steel and ∼65% for uncoated glass).

**FIG. 5.**
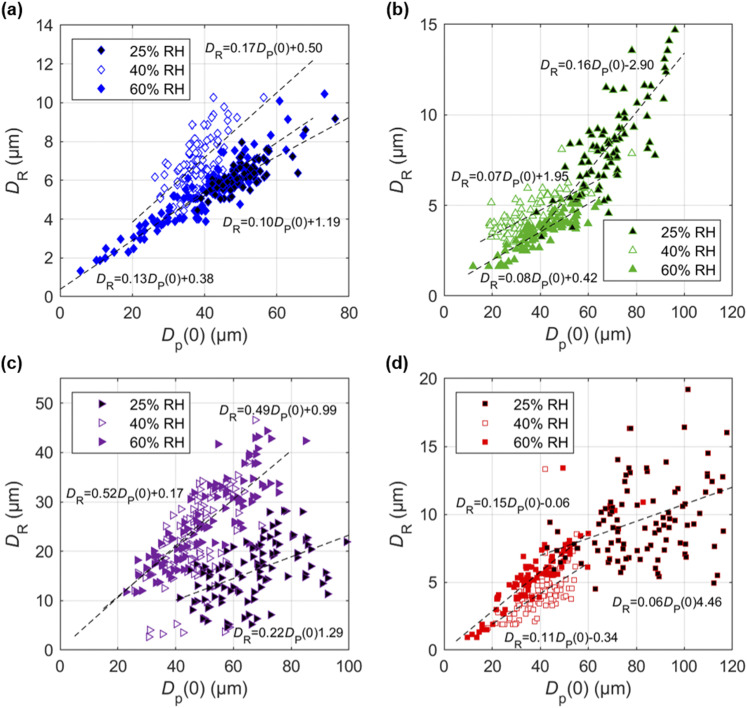
Variation in residue size *D*_R_ with the initial droplet size *D*_P_(0) at 22 °C and three humidity levels (25%, 40%, and 60% RH) on (a) coated glass substrate, (b) plastic, (c) stainless steel, and (d) uncoated glass substrates. Lines indicate linear least-squares fits to the data. For the stainless steel surface at 60% RH, the smaller residue size clusters are neglected when estimating the trend line.

The final residue size formed on each surface shows a dependence on the humidity level and the initial droplet size (this observation is consistent with Refs. 16 and 17) for all surfaces (Fig. 5). For the coated glass substrate [Fig. 5(a)], the residue size scales linearly with the initial droplet size at all humidity values with very similar slopes. Specifically, the minimum droplet size that can form a residue decreases with humidity, from ∼30 *µ*m at 25% RH to ∼5 *μ*m at 60% RH. We observe similar trends between the three humidity values for the other surfaces [Figs. 5(b)–5(d)]. At a fixed humidity level, the residue size scales linearly with the initial droplet size with a slope varying from ∼0.06 for uncoated glass to ∼0.22 for stainless steel at 25% RH and to ∼0.08 for plastic and ∼0.49 for stainless steel at 60% RH. The measurements on the stainless steel surface show the presence of two clusters that each scale differently with the initial droplet size at 60% RH. A cluster of large residues increases at a higher rate and a smaller cluster changes slowly with the initial droplet size. Note that we neglect the smaller size residues when estimating the linear trend line for stainless steel. We also observe a lower variation in the residue size at higher humidity (within each type of residue for stainless steel). Finally, the smallest droplets that form residues decrease with increasing humidity (from 25% RH to 60% RH), albeit to different levels. The coated glass surface shows the highest variation from ∼40 *µ*m at 25% RH to ∼5 *µ*m at 60% RH, followed by the remaining three surfaces that show a drop of ∼30 *µ*m changing from ∼40 *µ*m to ∼11 *µ*m, ∼12 *µ*m, and ∼10 *µ*m for the stainless steel, plastic, and uncoated glass surfaces, respectively.

## IV. CONCLUSION AND DISCUSSION

Overall, our findings provide a physical mechanism contributing to the long survival time and stability of viruses under practical settings. Specifically, we hypothesize that the residues with size 1–2 orders larger than those of SARS-CoV-2 found in our experiments can serve as a shield, insulating the virus against extreme environmental changes.^10,27^ This hypothesis is also supported by Bhardwaj and Agrawal^6^ and Corpet.^28^ Furthermore, the presence of a lipid bilayer with a hydrophilic outer surface on the virus^29^ allows them to remain stable in high humidity found within residues. Accordingly, the probability of forming residues and their stability can indicate the virus survivability on different surfaces. For instance, the residues are found to be much more difficult to form on copper, which shows the shortest survival time of SARS-CoV-2 in the work of van Doremalen *et al.*^4^ Compared with plastic, stainless steel has lower probability of sustaining the formed residue for long term at 40% RH, mirroring the survivability results for plastic and stainless steel reported in the work of van Doremalen *et al.*^4^

The physical insights gained from our work can be extended to other viruses that are transmitted through respiratory droplets (e.g., SARS/MERS viruses and flu viruses), particularly to SARS-CoV-1 that has a survivability trend very similar to those of SARS-CoV-2 on different surfaces.^4^ Our findings suggest that high temperature (through enhancing the evaporation rate) and low humidity can inhibit the formation of residues, lowering the survivability of viruses on surfaces. Regarding temperature effects, such inference is consistent with the reduced survivability of virus with increasing temperature reported in multiple studies.^3,30,31^ However, despite a number of studies investigating the humidity effect on virus survivability on surfaces,^30,31^ their experiments were conducted using virus-laden droplets of ∼mm size, which forms residues at all humidity conditions tested according to our study. Therefore, the probability of residue formation cannot be used to explain the variation of virus survivability with humidity in their studies, which are likely caused by other mechanisms. The adverse effect of humidity on virus infectivity reported in the literature^32,33^ points largely to airborne transmission, which can be explained by increased aerosol settling at higher humidity through condensation. In addition, there are a number of studies that investigated this effect based on statistical analyses of regional and global data.^34–36^ However, such studies, usually subject to various complex factors (e.g., differences in geography, culture, and policy), are difficult to be directly linked to the physical mechanism discussed in our study.

Our tests show that wiping with regular water-absorbent tissue paper can remove more than 95% of the residues on surfaces if disinfecting wipes are not available. Particularly, our results derived from the experiments using droplets with the size matching those generated during human breathing and speaking have specific implications for COVID-19, which display an exceedingly high rate of spread than earlier viruses, associated with high viral loads in the upper respiratory tract and potential transmission by asymptomatic/presymptomatic individuals.^37–39^ Our results suggest that even tiny droplets (<20 *μ*m) can leave residues under moderately high humidity (>40%) causing significant spread of the virus through surface contamination. Therefore, our study highlights the importance in wearing masks under such conditions toward minimizing the spread of the virus to surfaces through normal respiratory activities, e.g., breathing and speaking.^40^ In addition, lowering the indoor humidity when possible can suppress the formation of such residues (e.g., a significant drop in the fraction of residue forming droplets in steel below 15% RH and below 10% RH for other surfaces) and limit the spread of viral infection through contact from such small respiratory droplets, as we continue to reopen our economy and workplaces in the future.

In the end, we would also like to caution the readers from generalizing the quantitative results (e.g., evaporation rate and residue fraction) present in our experiments since they are dependent on specific surfaces and environmental conditions. Accordingly, it would be of practical significance to investigate the evaporation residues over a broader range of surface substrates and under different environmental factors (e.g., humidity and temperature), which can lead to actionable preventive measures to reduce the virus transmission through contaminated surfaces. Our work can potentially inspire a host of future research using more advanced diagnostic, analytical, and simulation tools to elucidate the formation and characteristics of residues and their connection with virus transmission.

## SUPPLEMENTARY MATERIAL

See the supplementary material for videos showing the process of droplet evaporation on different substrates.

## DATA AVAILABILITY

The data that support the findings of this study are available from the corresponding author upon reasonable request.
